# Optimization of Physical Refining Process of Camellia Oil for Reduction of 3-Monochloropropane-1,2-Diol (3-MCPD) Ester Formation Using Response Surface Methodology on a Laboratory Scale

**DOI:** 10.3390/molecules28083616

**Published:** 2023-04-21

**Authors:** Liqun Zhang, Pinggu Wu, Xiaoling Xiang, Dajin Yang, Liyuan Wang, Zhengyan Hu

**Affiliations:** 1 Center for Disease Control and Prevention of Hangzhou, Hangzhou 310021, China; mylittlebutterfl@126.com; 2 Zhejiang Provincial Center for Disease Control and Prevention, Hangzhou 310051, China; 3 Administration Bureau of Pidu Chinese Sichuan Cuisine Industrial Park, Chengdu 611730, China; 4 China National Center for Food Safety Risk Assessment, Beijing 100051, China

**Keywords:** 3-MCPD esters, response surface analysis, optimized refining process parameters, reduction of 3-MCPD

## Abstract

Refined and deodorized camellia oil has been reported to contain a high amount of 3-monochloropropane-1,2-diol esters (3-MCPDE) due to the high-temperature deodorization step. To reduce 3-MCPDE in camellia oil, the physical refining process of camellia oil was simulated on a laboratory scale. Response surface methodology (RSM) was designed to modify and optimize the refining process with five processing parameters (water degumming dosage, degumming temperature, activated clay dosage, deodorization temperature and deodorization time). The optimized new refining approach achieved a 76.9% reduction in 3-MCPDE contents, in which the degumming moisture was 2.97%, the degumming temperature was 50.5 °C, the activated clay dosage was 2.69%, the deodorizing temperature was 230 °C, and the deodorizing time was 90 min. A significance test and analysis of variance results demonstrated that the deodorization temperature and deodorization time contributed significantly to the reduction of 3-MCPD ester. The joint interaction effects of activated clay dosage and deodorization temperature were significant for 3-MCPD ester formation.

## 1. Introduction

Camellia oil is one of the most important edible oils in China; it is rich in oleic acid content and many natural antioxidants with various biological activities [1]. Camellia oil is not only used for cooking, but applied as functional food for lowering blood pressure and suppressing the occurrence of asthma [2]. However, refined camellia oils exhibited the highest contents of 3-MCPDE in a survey of 143 edible oils and fats collected from Chinese markets [3]. Recent research has suggested that the intrinsic components of the oil itself could be the precursors to 3-MCPD ester formation [4,5], and the reduction of 3-MCPD ester in camellia oil has been studied relatively little so far. It is of vital importance to study the reduction of 3-MCPD ester in camellia oil, as China is the largest producer and exporter of Camellia oil in the world.

The chloropropanol ester pollution has been one of the most pressing food safety issues in the world in recent years. All kinds of edible oils and hot processed foods contain various chloropropanol esters [6]. The free forms of Chloropropanol esters are chloropropanol compounds. Chloropropanols include monochloropanol and dichloropanol, which are recognized as food-processing pollutants. It has been reported that the amount of monochloropanol produced during food processing is usually 100 to 10,000 times that of dichloropanol, and the content of 3-Chloro-1,2-Propanediol (3-MCPD) is usually several to 10 times that of 2-chloro-1,3-propanediol [7]. Therefore, the detection of 3-MCPD content in food can roughly reflect the production of chloropropanols in food processing. Similarly, the pollution of 3-MCPD ester is the most prominent in all kinds of food. In a study of chloropropyl ester, researchers also mainly studied the content, formation mechanism and toxicity of 3-MCPD ester. People now pay more and more attention to 3-MCPD esters, and their research is also becoming more and more in-depth [8,9,10].

The International Agency for Research on Cancer (IARC) has assessed the toxicity of 3-MCPD, which was classified as possibly carcinogenic to humans (Group 2B) [11]. Vegetable fats and oils were identified as one of the major contributors to dietary exposure of 3-MCPD by an exposure assessment by the European Food Safety Authority [12]. The Scientific Committee on Food (SCF) and the Joint FAO/WHO Expert Committee on Food Additives (JECFA) have established a tolerable daily intake (TDI) of 2 µg/kg body weight for free 3-MCPD [13]. In 2016, the Panel on Contaminants in the Food Chain concluded that a TDI of 0.8 µg/kg body weight would be suitable for free and esterified 3-MCPD [14].

Great efforts have been devoted to the reduction of 3-MCPD during the physical refining process of oil due to their potential toxicity [15,16]. Different strategies are used, including removal of chloroester precursors from the raw material prior to the deodorization step [5], changing of the refining process or potential inhibitors of 3-MCPD esters from the refined product [17]. Previously, studies indicated that appearance of MCPDE was directly linked to exposure to high temperatures, and thus the deodorization (water steam distillation of volatile substances) step of the oils [18]. These processes liberate the source of protons and supply sufficient energy for the 3-MCPD ester formation to take place [19,20,21].

In the refining process of edible oils, response surface methodology (RSM) proved to be a powerful tool for developing, improving, and optimizing the process design in lipid studies [22,23]. The mathematical model is designed to describe the response of interest influenced by several variables, to build models and thus search optimum process conditions to achieve the least 3-MCPD ester formation [24,25]. Factors such as water degumming dosage, degumming temperature, activated clay dosage, deodorization temperature and deodorization time are responsible for the 3-MCPD content in refined oils.

In this paper, a promising method based on response surface methodology (RSM) for reduction of 3-MCPD esters in the physical refining procedures of camellia oil was proposed. The role of various factors that can influence the level of 3-MCPD esters during refining processing of camellia oil was extensively studied. Additionally, the optimized refining process parameters were obtained, which successfully achieved minimum 3-MCPD ester formation on a laboratory scale.

## 2. Results

### 2.1. The Influence of Different Refining Processes on the Formation of 3-MCPD Ester

**Figure 1:**
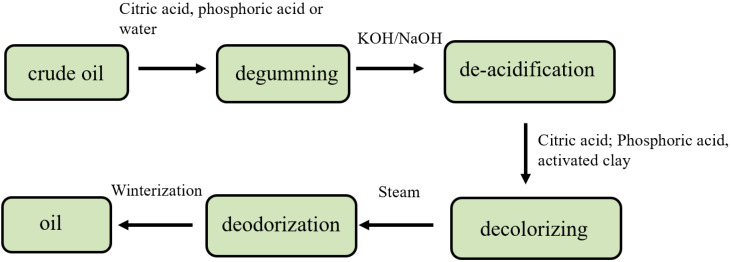
Flow chart of the camellia oil refining process.

**Figure 2:**
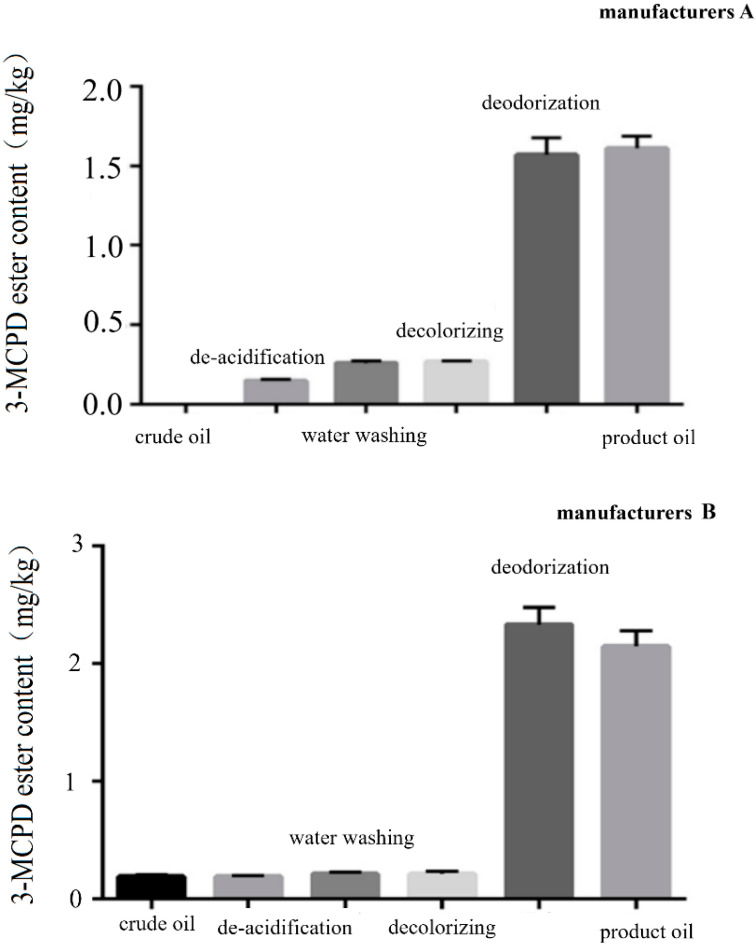

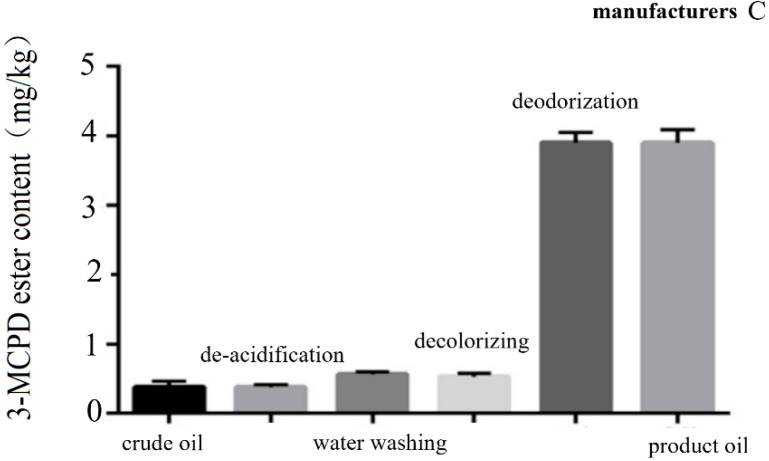
The influence of different refining processes on the formation of 3-MCPD ester, measured in the camellia oil refining process of three different manufacturers (**A**–**C**).

The flow chart of the camellia oil refining process is shown in Figure 1. The measurement results of 3-MCPD ester in camellia oil samples from three camellia oil refineries at different stages are shown in Figure 2. The results show that the refining process of camellia oil has an important influence on the formation of 3-MCPD ester, and the content of 3-MCPD ester in camellia oil produced by different processes of three manufacturers is greatly different. The crude camellia oil samples of three manufacturers contain little or no 3-MCPD ester, and the content of 3-MCPD ester involved in alkali-refining, water-washing and decoloring oil samples is less than 0.50 mg/kg. However, the content of 3-MCPD ester in the three deodorized oils is very high. The content of 3-MCPD ester in deodorized oils from manufacturers A, B and C is 1.61 mg/kg, 2.15 mg/kg and 3.89 mg/kg, all exceeding 1.0 mg/kg. This demonstrated that the deodorization process contributed significantly to the formation of 3-MCPD esters. Therefore, the influence of deodorization temperature as well as deodorization time on the formation of 3-MCPD ester was systematically studied.

### 2.2. Design of the 3-MCPD Ester Formation Model Based on RSM

The experimental design of five variable and five level response surface methodology analysis is listed in Table 1, and 32 experimental settings are presented in Table 2. The data from each response were fitted with the factors by multiple regression to a second-order model polynomial equation, and the developed models were as follows (Equation (1)):

(1) $$Y=3.62-0.095X_{1}+0.14X_{2}-0.22X_{3}+0.79X_{4}+0.35X_{5}-0.14X_{1}X_{3}-0.12X_{1}X_{5}\;+0.2X_{3}X_{4}-0.14X_{4}X_{5}-0.094{X_{1}}^{2}-0.19{X_{4}}^{2}-0.49{X_{5}}^{2}$$

where *Y* is the response, and *X*_1_, *X*_2_, *X*_3_, *X*_4_ and *X*_5_ are independent variables. *X*_1_ is the water degumming dosage (*X*_1_, 0.75–3.75%), *X*_2_ is the degumming temperature (*X*_2_, 40–80 °C), *X*_3_ is the activated clay dosage (*X*_3_, 0.5–3.5%), *X*_4_ is the deodorization temperature (*X*_4_, 210–290 °C), and *X*_5_ is the deodorization time (*X*_5_, 1–3 h). Subsequently, using numerical optimization, the responses of interest from the developed models were overlaid and optimized for the lowest 3-MCPD content and acceptable oil quality.

### 2.3. RSM Analysis

A significance test and analysis of variance (ANOVA) were applied to the regression equation. The ANOVA for response surface quadratic model is listed in Table 3. The polynomial equation is of significant regression, with *p* < 0.0001. The lack-of-fit test was 0.0973, which is not significant. The predictive capabilities of the models are expressed by the coefficients of determination, R^2^, which were 0.9839, close to the calibration factor of 0.9546. The results indicated that the models accurately represent the data in the experimental regions [10].

### 2.4. Effects of Processing Parameters on the Formation of 3-MCPD Ester

As shown in Table 3, the greatest 3-MCPD ester reduction was significantly contributed by the deodorization temperature (*X*_4_, *p* < 0.0001), and deodorization time (*X*_5_, *p* < 0.0001). This is consistent with previous statistical analysis and modeling results that temperature of the steam distillation was highly influential on bound 3-MCPD and glycidyl ester formation [25]. High temperature has the effect of promoting 3-MCPD ester formation. The degumming temperature (*X*_2_, *p* < 0.01) and activated clay dosage (*X*_3_, *p* < 0.01) also showed a significant role, but to a lesser extent. The water degumming dosage (*X*_1_, *p* < 0.05) also has significant role in the formation of 3-MCPD ester, but to a much lesser extent. This indicated that all the five factors play important roles in the 3-MCPD ester formation during the refining process of camellia oil, among which deodorization temperature (*X*_4_) and deodorization time (*X*_5_) contributed the most.

However, the five factors did not act separately. The joint efforts of these factors must be considered. The interaction effects of different factors were examined using the generated response surface plots of 3-MCPD ester content. The joint interaction effects of activated clay dosage and deodorization temperature (*X*_3_*X*_4_) contributed most significantly to the 3-MCPD ester formation (*p* < 0.01). The joint effect of the water degumming dosage and activated clay dosage (*X*_1_*X*_3_), the water degumming dosage and deodorization time (*X*_1_*X*_5_), and the deodorization temperature and deodorization time (*X*_4_*X*_5_) also showed significant effect on the 3-MCPD ester formation (*p* < 0.05), but to a lesser extent. However, the interaction effects of degumming temperature and activated clay dosage (*X*_2_*X*_3_) and the degumming temperature and deodorization temperature (*X*_2_*X*_4_) showed no significant influence on the reduction of 3-MCPD ester (*p* > 0.05). The quadratic terms *X*_4_^2^ and *X*_5_^2^ have an extremely significant influence on the 3-MCPD ester formation, while *X*_1_^2^ has a significant influence.

#### 2.4.1. Joint Interaction Effects of Activated Clay and Deodorization Temperature (*X*_3_*X*_4_) on 3-MCPD Ester Reduction

**Figure 3:**
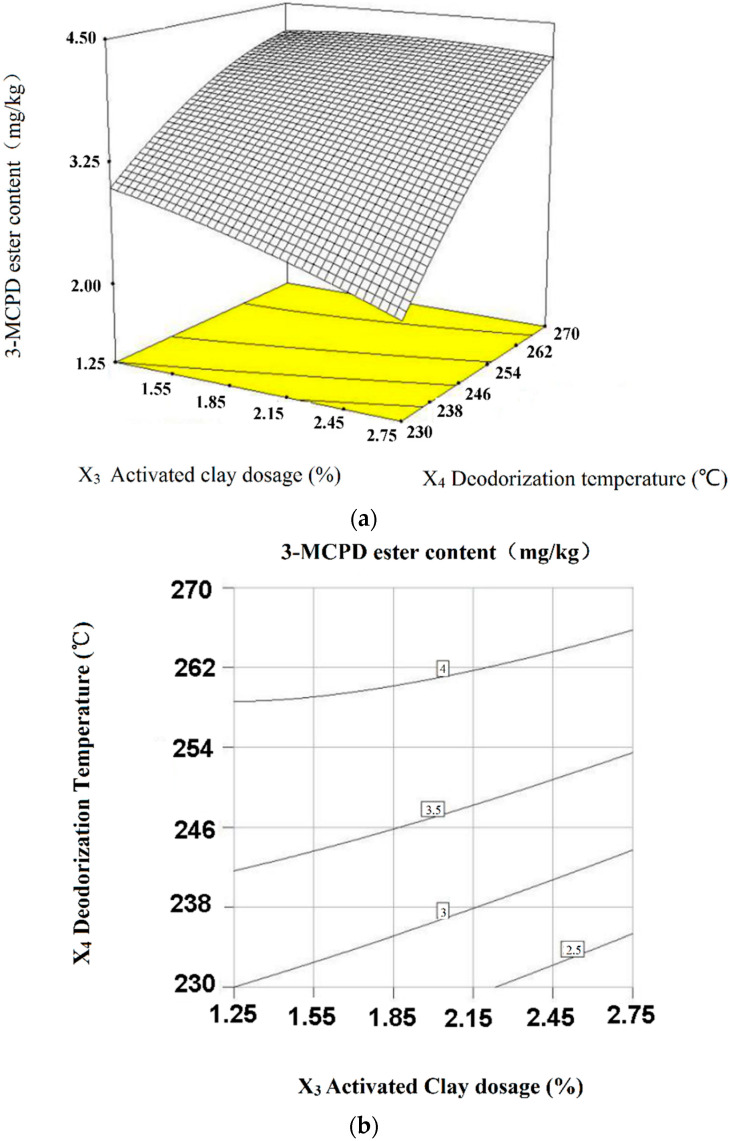
Response surface curves (**a**) and contours (**b**) of activated clay and deodorization temperature towards 3-MCPD ester (interactions of *X*_3_*X*_4_, *p* < 0.01).

As shown in the contour map of Figure 3, when the deodorization temperature (*X*_4_) is certain, with the increase in activated clay dosage (*X*_3_), the content of 3-MCPD ester decreases. This indicates that the activated clay removed 3-MCPD ester precursors; when the amount of activated clay increases, more precursors can be removed from the crude oil, and hence a greater reduction of 3-MCPD ester occurs. The most effective factor that influences the formation of 3-MCPD esters in refined oils is deodorization temperature [26,27]. While the activated clay dosage (*X*_3_) is certain, with the decrease in the deodorization temperature (*X*_4_), the amount of 3-MCPD ester decreases. Deodorization at temperatures below 240 °C seems to be advantageous to achieving low concentrations of contaminants [28]. Apparently, the level of 3-MCPD esters increases with the rise of temperature, as shown in Figure 3a. High temperature may favor the formation of the glycidyl esters (GE). The formed GE are not volatile under these conditions and stay in the system. Nevertheless, an increased hydrolysis of triacylglycerides (TG) may lead to a higher precursor concentration of monoacylglycerides (MG) and diacylglycerides (DG) forming 3-MCPDE [28]. However, as the deodorization temperature increases further, the trend of the increase in the level of 3-MCPD esters becomes less apparent; this we may attribute to the degradation of 3-MCPD esters involved in isomerisation, dechlorination and deacylation reactions [29], associated with high temperature. This is consistent with the results of lab-scale investigations of physical refining processes and their effects on the formation of 3-monochloropropane-1,2-diol (3-MCPD) esters in peanut oil [30].

This result confirms the importance of controlling the activated clay dosage during crude oil degumming. Both increasing the activated clay dosage and decreasing the deodorization temperature may lead to reduction of 3-MCPD ester.

#### 2.4.2. Joint Interaction Effects of Degumming Water and Activated Clay (*X*_1_*X*_3_) on 3-MCPD Ester Reduction

**Figure 4:**
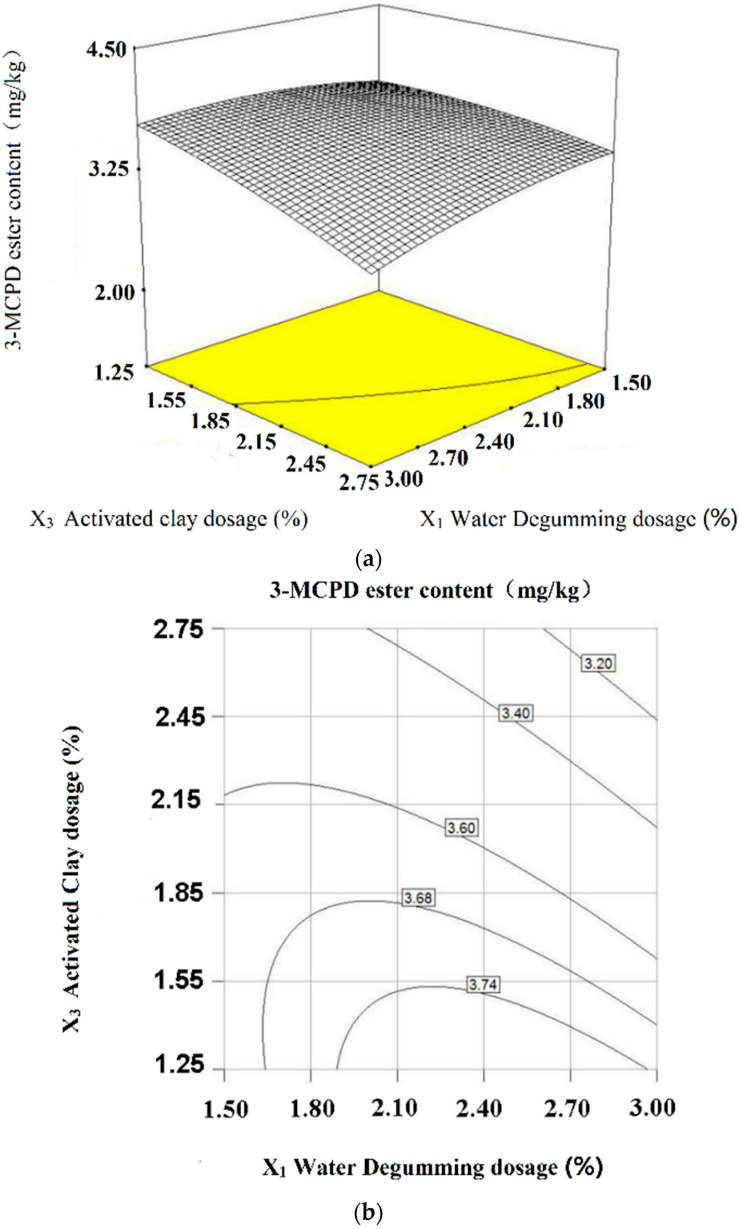
Response surface curves (**a**) and contours (**b**) of degumming water and activated clay towards on 3-MCPD ester (interactions of *X*_1_*X*_3_, *p* < 0.05).

The three-dimensional response surface and contour maps were plotted based on regression equation and variance analysis, in which the interaction of two factors can be directly seen. The response surface curves and contours of degumming water (*X*_1_) and activated clay (*X*_3_) towards 3-MCPD ester were illustrated in Figure 4. When the degumming water dosage (*X*_1_) is certain, with the increase in the activated clay dosage (*X*_3_), the 3-MCPD ester content decreases. The activated clay is used to remove impurities, such as oxidative products, phosphorus, trace metals and color pigments, before deodorizing the oil. This reduction may be attributed to the adsorption of activated clay, which removed the precursors of 3-MCPD formation. The high amount of activated clay was thought to be beneficial for the greater reduction of 3-MCPDE and GE [14,28]. Meanwhile, when the activated clay dosage (*X*_3_) is certain, with the increase in water dosage (*X*_1_), the 3-MCPD ester content decreases.

#### 2.4.3. Joint Interaction Effects of Degumming Water and Deodorization Time (*X*_1_*X*_5_) on 3-MCPD Ester Reduction

**Figure 5:**
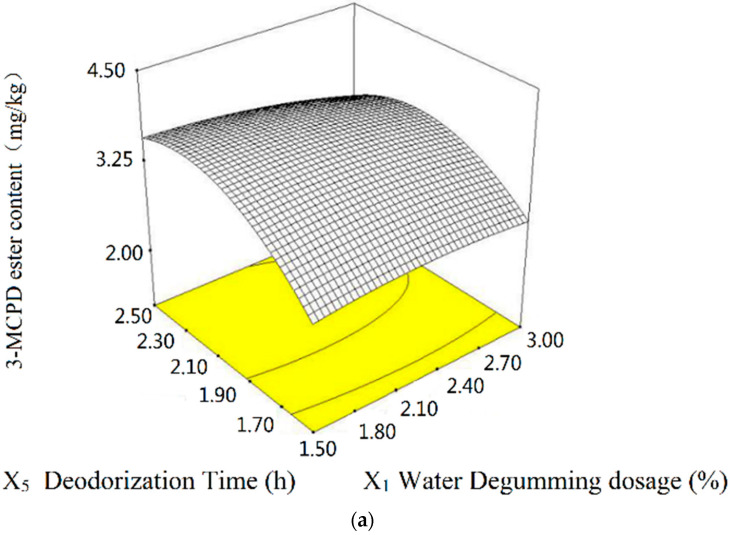

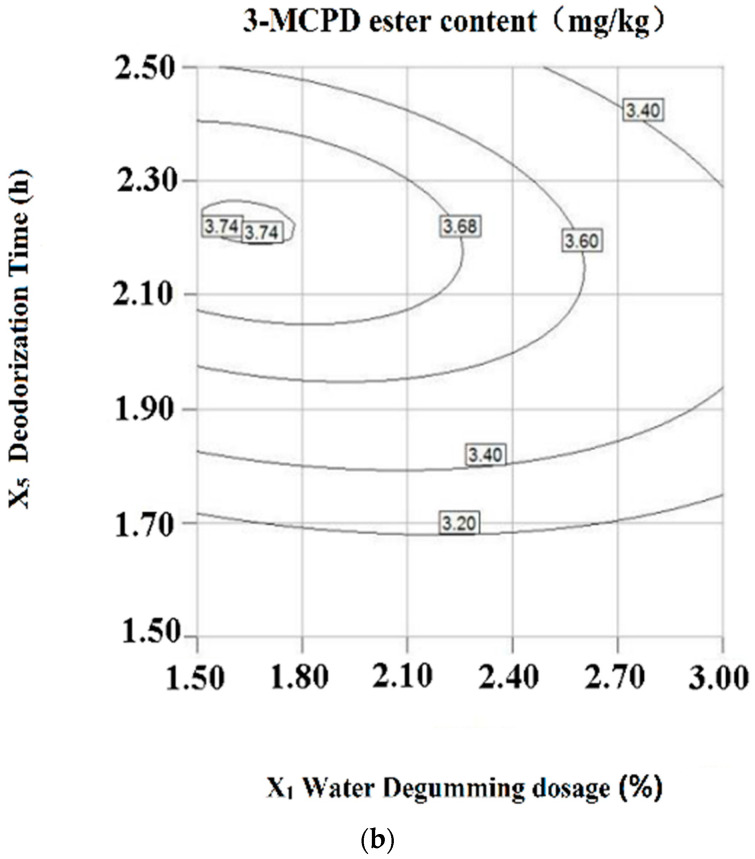
Response surface curves (**a**) and contours (**b**) of degumming water and deodorization time towards 3-MCPD ester (interactions of *X*_1_*X*_5_, *p* < 0.05).

In the response surface and contour map of Figure 5, the relationship of degumming water (*X*_1_) and deodorization time (*X*_5_) with the formation of 3-MCPD ester is demonstrated. When the deodorization time (*X*_5_) is certain, with the increase in degumming water dosage (*X*_1_), the formation of 3-MCPD ester was greatly reduced, which could be primarily based on the elimination of 3-MCPD ester precursors with greater amounts of degumming water dosage [22]. When the degumming water dosage (*X*_1_) is certain, with the increase in the deodorization time (*X*_5_), the content of 3-MCPD ester increased first and then decreased. This may be because the longer the time taken for deodorization, the greater the dissolution rate of 3-MCPD ester compared with the rate of their formation [31].

#### 2.4.4. Joint Interaction Effects of Deodorization Time and Deodorization Temperature (*X*_4_*X*_5_) towards 3-MCPD Ester

**Figure 6:**
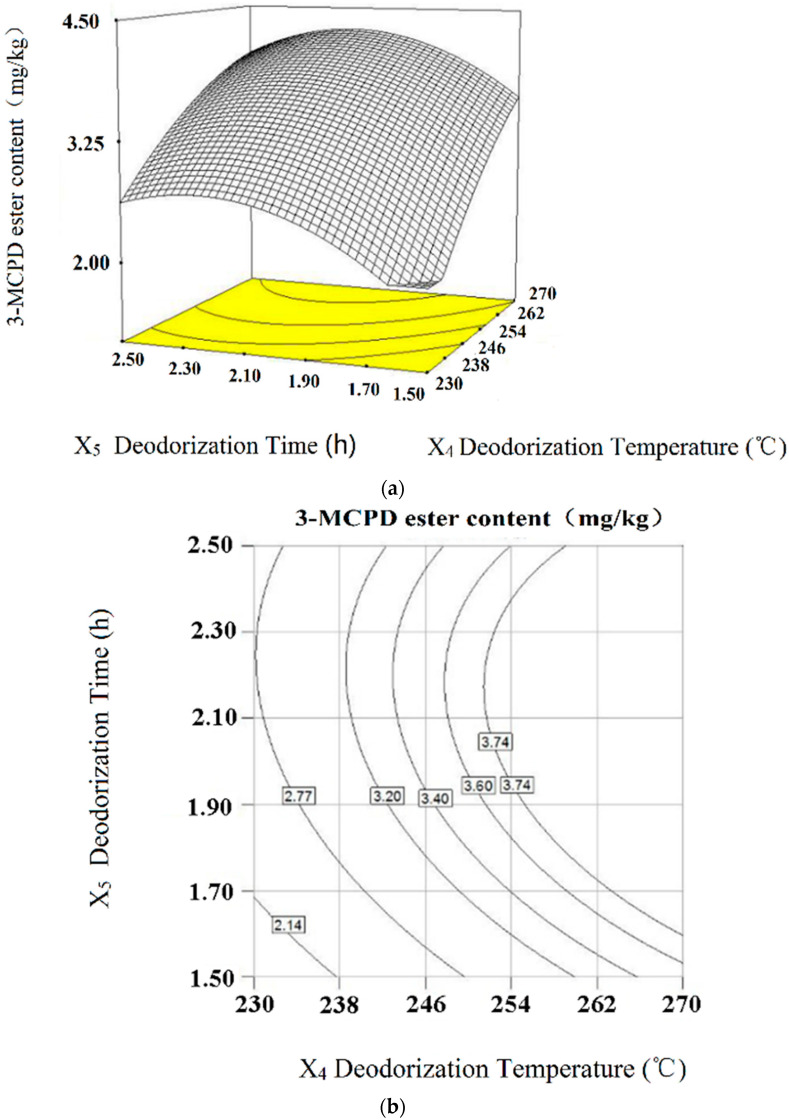
Response surface curves (**a**) and contours (**b**) of deodorization time and deodorization temperature towards 3-MCPD ester (interactions of *X*_4_*X*_5_, *p* > 0.05).

The response surface curves and contours of deodorization time and deodorization temperature towards 3-MCPD ester (Interactions of *X*_4_*X*_5_, *p* > 0.05) are shown in Figure 6. The interactions of deodorization time (*X*_4_) and deodorization temperature (*X*_5_) are very apparent; when the deodorization time is certain, the content of 3-MCPD ester formation increases with the increase in deodorization temperature (*X*_5_). The increase in temperature during the deodorization process may cause greater activation of the precursors and supply sufficient energy for 3-MCPD ester formation. When the deodorization temperature (*X*_5_) is certain, the formation of 3-MCPD ester increases first and then decreases with the increase in deodorization time (*X*_4_).

### 2.5. Model Evaluation

**Figure 7:**
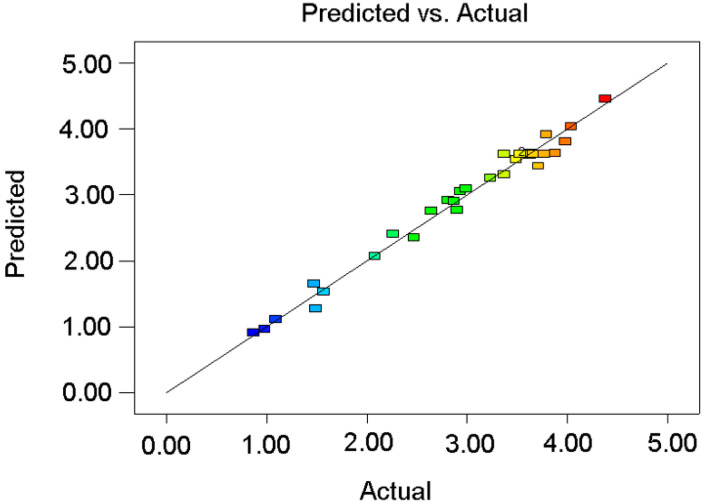
Goodness-of-fit of predictive and actual values of the polynomial model.

The goodness-of-fit of the predictive and actual value of the polynomial model are presented in Figure 7. The predicted values are in linear correlation with the practical experimental values, which indicated the adequacy and reliable of predictive capabilities of the models established by RSM.

## 3. Discussion

The optimum conditions for the reduction of 3-MCPD ester formation in refined Camellia oil can be obtained by RSM analysis. The steady point as well as the minimum value was found, and the corresponding factors are *X*_1_ = 2.97%, *X*_2_ = 50.49 °C, *X*_3_ = 2.69%, *X*_4_ = 230.06 °C, and *X*_5_ = 1.52 h, and the predicted estimation of 3-MCPD esters content was 0.87 mg/kg. In order to verify the model’s predicted value with that of the real experiment value, practical experiment of the refining process was conducted according to the optimized experimental conditions. Taking into account the practical experimental conditions, the adjusted experimental conditions were a 2.97% water dosage, a 50.5 °C degumming temperature, a 2.69 % bleaching clay dosage, a 230 °C deodorization temperature, and a deodorization time of 90 min, producing the minimum content of 3-MCPD esters, at about 0.83 mg/kg. The practical experimental value was very close to the value predicted by the model, with a difference of −10%. The optimization can be considered valid and the model of our study acceptable when the average difference is lower than 15%. Therefore, the model developed in this study correlates well with the practical experiment. Simultaneously, the optimized model gave a 76.9% reduction in 3-MCPD esters when conducting the refining process, compared with the initial experimental parameters, with a content of 3-MCPD esters of 3.60 mg/kg.

## 4. Materials and Methods

### 4.1. Samples and Reagents

Three crude camellia oil samples were supplied by three different Camellia oil manufacturers (named A, B, and C) of Changshan City of Zhejiang Province (Changshan, China). Bis-1,2-palmitoyl-3-chloropropanediol (PP-3-MCPD), and bis-1,2-palmitoyl-3-chloropropanediol-d5 (PP-d5-3-MCPD) was purchased from Toronto Research Chemicals Inc. (Toronto, ON, Canada) at purities > 98%. Chromatography grade n-hexane, ethyl acetate, acetone and diethyl ether were purchased from J. T. Baker^®^ Chemicals (Radnor, PA, USA). Chromatography grade methyl tert-butyl ether, methanol, isooctane and analytical grade sodium methoxide were purchased from Aladdin Reagent (Shanghai, China). Sodium bromide, phenylboronic acid and anhydrous sodium sulfate (used after baking at 450 °C for 4 h) were purchased from Sinopharm Chemical Reagent Co., Ltd (Shanghai, China). The (n-propyl)ethylenediamine (PSA) (6 cc, 200 mg) SPE columns were obtained from Waters (Milford, MA, USA).

### 4.2. The Refining Process of Camellia Oil

**Figure 8:**
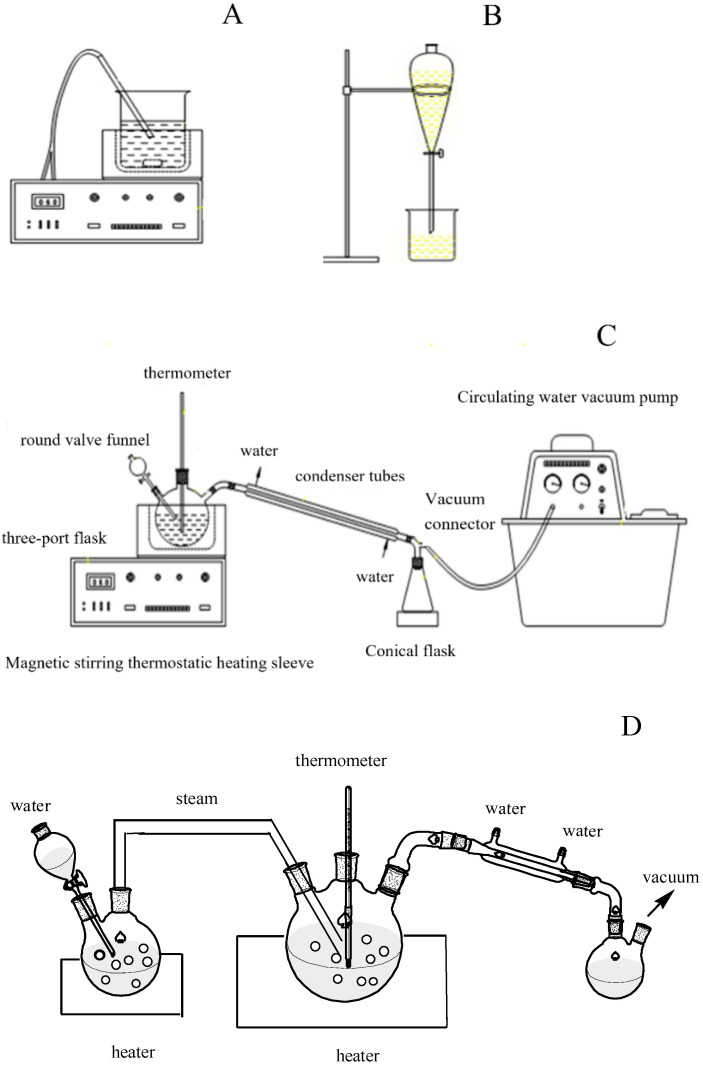
Device diagram of simulated refining experiment in the laboratory. (**A**) degumming and de-acidification; (**B**) water washing; (**C**) decolorizing; and (**D**) deodorization.

The device diagrams of simulated refining experiments of crude camellia oil in laboratory are shown in Figure 8.

#### 4.2.1. The Degumming Process

Approximately 200 g of crude camellia oil was weighed into a 1 L laboratory-scale refining flask with a stirrer and heating mantle. A certain volume of hot water was put into the oil. The oil was stirred and heated for 30 min, and was then centrifugated at 10,000 r/min for 10 min. To remove free fatty acid in the crude oil, sodium hydroxide was added into the water-degummed camellia oil and heated from 30 °C to 60 °C. After that, the oil was left still for 24 h, and the upper layer was separated for the decolorization process.

#### 4.2.2. The Decolorization Process

A certain amount of water-degummed camellia oil was weighed, and a certain amount of activated clay was added and heated at 90 °C in a vacuum of −0.1 MPa. The oil was then stirred for 30 min and centrifugated at 10,000 r/min for 10 min.

#### 4.2.3. The Deodorization Process

Steam distillation runs were carried out using laboratory-scale distillation equipment composed of a glass flask, magnetic stirring heating apparatus and vacuum pumps, which simulated the steam distillation decolorization process in the oil manufactory. Water was vaporized on a heated glass surface and transferred into an oil sample through a perforated glass tube. The boiling effect was achieved by introducing the steam into heated oil with the help of a vacuum, as shown in Figure 8D.

### 4.3. Experimental Design and Response Surface Methodology Analysis

The refining process of camellia oil was influenced by many factors during the producing process, among which five factors contributed significantly, based on the actual technical parameters of the manufacturers [17]. The five factors and their five levels were the water degumming dosage (*X*_1_, 0.75–3.75%), the degumming temperature (*X*_2_, 40–80 °C), the activated clay dosage (*X*_3_, 0.5–3.5%), the deodorization temperature (*X*_4_, 210–290 °C), and the deodorization time (*X*_5_, 1–3 h).

With the yield of 3-MCPD ester as an indicator, the primary and secondary relationships of various factors were determined. The central composite rotatable design (CCRD) in Design Expert V8.0.6 software (Stat-Ease, Inc., Minneapolis, MN, USA) was used to fit the experimental data, establish a quadratic regression equation, draw a response surface, and use the F-test method for significance analysis to describe the formation law of 3-MCPD ester in the refining process. The optimal refining parameters that produced the smallest yield of 3-MCPD ester in camellia oil were selected.

Using Design Expert V8.0.6 software, with water degumming dosage, degumming temperature, active clay content, deodorization temperature, and deodorization time as response variables, and with 3-MCPD ester as response value (index), the experiment was conducted according to the experimental plan shown in Table 2, consisting of a total of 32 experiments. The quadratic polynomial regression equation of the experimental results obtained was fitted, and the prediction model was obtained.

### 4.4. Model Verification and Statistical Analysis

The data from the RSM were statistically analyzed using SPSS 20.0 (IBM, New York, NY, USA). A significance test and analysis of variance (ANOVA) were applied to the regression equation. All the samples were analyzed three times in parallel, and the results are presented by mean values ± standard deviation (mean ± SD). By multiple regression to a second-order model polynomial equation, the data from each response were fitted as follows (Equation (2)):

(2) $$y=\beta_{0}+\sum_{i=1}^{5}\beta_{i}X_{i}+\sum_{i=1}^{5}\beta_{ii}X_{i}^{2}+\sum_{i=1}^{5}\sum_{j=i+1}^{4}\beta_{ij}X_{i}X_{j}$$

where y is the response; *β*_0_, *β*_i_, *β_ii_*, and *β_ij_* are the constant coefficients of the intercept, linear, quadratic, and interaction terms, respectively; and *X_i_* and *X_j_* are independent variables. The insignificant (*p* > 0.05) factors and the interactions between them were removed. The lowest 3-MCPD contents of the refining process were optimized by numerical optimization by the developed models.

### 4.5. 3-MCPD Ester Determination Using GC-MS

The 3-MCPD ester contents of refined camellia oil were determined using the method previously developed by us [32] and using an Agilent (Palo Alto, CA, USA) Model 5975C gas chromatograph coupled with a Model 6890 mass spectrometer. The column used was a DB-5 MS capillary column (30 m long and 0.25 mm in diameter with a film thickness of 0.25 µm; Agilent).

### 4.6. Oil Quality Analysis

The oil acid value, peroxide value, and Lovibond color of crude camellia oil and refined camellia oil of each run were determined according to National Food Safety Standard of China (GB/T2016) 229 (National Health Commission of the People’s Republic of China, 2016) [33], (GB/T2016) 227 (National Health Commission of the People’s Republic of China, 2016) [34], and the National Standard of China (GB/T 22460-2008) [35], respectively.

## 5. Conclusions

In this study, the physical refining process of camellia oil, particularly the pretreatment steps, was modified and therefore optimized for the reduction of 3-MCPD ester formation, using response surface methodology. The optimized refining process parameters for minimum 3-MCPD ester production were a degumming moisture of 2.97%, a degumming temperature of 50.5 °C, an activated clay dosage of 2.69%, a deodorizing temperature of 230 °C, and a deodorizing time of 90 min. The optimized model gave a 76.9% reduction in 3-MCPD esters in a laboratory-scale refining process, compared with the initial experimental parameters. The accuracy of the model was verified by the practical experimental data. The results may provide new insight into the mechanism of formation of 3-MCPD esters and feasible approaches for reduction of 3-MCPD esters in camellia oil, in a low-cost manner, for industry production.

## Figures and Tables

**Table 1:** The experimental design of five variable and five level RSM.

Level	Fators				
	*X*_1_ (Water Degumming Dosage, %)	*X*_2_ (Degumming Temperature, °C)	*X*_3_ (Activated Clay Dosage, %)	*X*_4_ (Deodorization Temperature, °C)	*X*_5_ (Deodorization Time, h)
−2	0.75	40	0.5	210	1
−1	1.50	50	1.25	230	1.5
0	2.25	60	2.00	250	2
1	3.00	70	2.75	270	2.5
2	3.75	80	3.50	290	3

**Table 2:** Arrangement and results of the response surface design.

	*X*_1_ (Water Degumming Dosage, %)	*X*_2_ (Degumming Temperature, °C)	*X*_3_ (Activated Clay Dosage, %)	*X*_4_ (Deodorization Temperature, °C)	*X*_5_ (Deodorization Time, h)	3-MCPD Ester Content (mg/kg)
1	2.25 (0)	80.00 (2)	2.00 (0)	250.00 (0)	2.00 (0)	3.88
2	1.50 (−1)	70.00 (1)	2.75 (1)	270.00 (1)	1.50 (−1)	3.49
3	3.00 (1)	70.00 (1)	1.25 (−1)	270.00 (1)	1.50 (−1)	3.63
4	1.50 (−1)	70.00 (1)	1.25 (−1)	270.00 (1)	2.50 (1)	3.79
5	2.25 (0)	60.00 (0)	2.00 (0)	250.00 (0)	2.00 (0)	3.77
6	2.25 (0)	60.00 (0)	2.00 (0)	250.00 (0)	2.00 (0)	3.66
7	1.50 (−1)	50.00 (−1)	1.25 (−1)	230.00 (−1)	2.50 (1)	2.64
8	1.50 (−1)	50.00 (−1)	1.25 (−1)	270.00 (1)	1.50 (−1)	3.23
9	1.50 (−1)	70.00 (1)	1.25 (−1)	230.00 (−1)	1.50 (−1)	1.47
10	3.00 (1)	70.00 (1)	1.25 (−1)	230.00 (−1)	2.50 (1)	2.81
11	2.25 (0)	60.00 (0)	0.50 (−2)	250.00 (0)	2.00 (0)	3.98
12	2.25 (0)	40.00 (−2)	2.00 (0)	250.00 (0)	2.00 (0)	2.99
13	2.25 (0)	60.00 (0)	2.00 (0)	250.00 (0)	2.00 (0)	3.61
14	3.00 (1)	50.00 (−1)	2.75 (1)	230.00 (−1)	2.50 (1)	1.57
15	3.00 (1)	50.00 (−1)	2.75 (1)	270.00 (1)	1.50 (−1)	2.9
16	2.25 (0)	60.00 (0)	2.00 (0)	250.00 (0)	2.00 (0)	3.66
17	1.50 (−1)	50.00 (−1)	2.75 (1)	270.00 (1)	2.50 (1)	4.04
18	3.00 (1)	70.00 (1)	2.75 (1)	270.00 (1)	2.50 (1)	3.63
19	2.25 (0)	60.00 (0)	2.00 (0)	250.00 (0)	2.00 (0)	3.37
20	0.75 (−2)	60.00 (0)	2.00 (0)	250.00 (0)	2.00 (0)	3.71
21	3.00 (1)	50.00 (−1)	1.25 (−1)	230.00 (−1)	1.50 (−1)	2.08
22	2.25 (0)	60.00 (0)	2.00 (0)	210.00 (−2)	2.00 (0)	1.49
23	2.25 (0)	60.00 (0)	2.00 (0)	250.00 (0)	1.00 (−2)	0.98
24	3.00 (1)	70.00 (1)	2.75 (1)	230.00 (−1)	1.50 (−1)	1.09
25	2.25 (0)	60.00 (0)	3.50 (2)	250.00 (0)	2.00 (0)	2.87
26	1.50 (−1)	70.00 (1)	2.75 (1)	230.00 (−1)	2.50 (1)	2.26
27	2.25 (0)	60.00 (0)	2.00 (0)	250.00 (0)	2.00 (0)	3.53
28	2.25 (0)	60.00 (0)	2.00 (0)	250.00 (0)	3.00 (2)	2.47
29	1.50 (−1)	50.00 (−1)	2.75 (1)	230.00 (−1)	1.50 (−1)	0.87
30	3.00 (1)	50.00 (−1)	1.25 (−1)	270.00 (1)	2.50 (1)	3.37
31	2.25 (0)	60.00 (0)	2.00 (0)	290.00 (2)	2.00 (0)	4.38
32	3.75 (2)	60.00 (0)	2.00 (0)	250.00 (0)	2.00 (0)	2.93

**Table 3:** ANOVA for response surface quadratic model.

Sources of Variation	Sum of Squares	Degree of Freedom	Mean Square	F Value	*p* Value
Model	29.54	20	1.48	33.56	<0.0001 **
*X*_1_	0.21	1	0.21	4.88	0.0493 *
*X*_2_	0.44	1	0.44	10.00	0.0090 **
*X*_3_	1.21	1	1.21	27.50	0.0003 **
*X*_4_	15.15	1	15.15	344.26	<0.0001 **
*X*_5_	2.89	1	2.89	65.69	<0.0001 **
*X*_1_ *X*_2_	0.064	1	0.064	1.45	0.2540
*X*_1_ *X*_3_	0.31	1	0.31	7.06	0.0223 *
*X*_1_ *X*_4_	0.11	1	0.11	2.51	0.1413
*X*_1_ *X*_5_	0.25	1	0.25	5.62	0.0371 *
*X*_2_ *X*_3_	0.032	1	0.032	0.72	0.4155
*X*_2_ *X*_4_	0.018	1	0.018	0.40	0.5406
*X*_3_ *X*_4_	0.66	1	0.66	15.00	0.0026 **
*X*_4_ *X*_5_	0.30	1	0.30	6.81	0.0243 *
*X*_1_^2^	0.26	1	0.26	5.86	0.0340 *
*X*_2_^2^	0.12	1	0.12	2.82	0.1215
*X*_3_^2^	0.13	1	0.13	3.04	0.1093
*X*_4_^2^	1.06	1	1.06	24.06	0.0005 **
*X*_5_^2^	7.11	1	7.11	161.65	<0.0001 **
Residual error	0.48	11	0.044		
Lack of fit	0.39	6	0.065	3.46	0.0973
Purely random error	0.094	5	0.019		
Overall error	30.02	31			

Note: *: *p* < 0.05, significant; **: *p* < 0.01, extremely significant.

## Data Availability

The data that support the findings of this study are available from the corresponding author, upon reasonable request.
